# Supplementary material for: Multiscale Analysis and Validation of Effective Drug Combinations Targeting Driver KRAS Mutations in Non-Small Cell Lung Cancer
Source: Int J Mol Sci. 2023 Jan 5;24(2):997. doi: 10.3390/ijms24020997 (PMC9867122; doi:10.3390/ijms24020997)
Supplement: Supplementary file 1 [file ijms-24-00997-s001.zip › SI.pdf]

International Journal of
Molecular Sciences

MDPI

Article

Supplementary Material: Multiscale Analysis and Validation of Effective Drug Combinations Targeting Driver Kras Mutations in Non-Small Cell Lung Cancer

Liana Bruggemann [1], Zackary Falls [1], William Mangione [1], Stanley A Schwartz [2], Sebastiano Battaglia [3], Ravikumar Aalinkeel [2], Supriya D. Mahajan [2,*], Ram Samudrala [1,*]

1 Department of Biomedical Informatics, University at Buffalo
2 Department of Medicine, University at Buffalo
3 Roswell Park Cancer Institute, Buffalo NY
* Correspondence: smahajan@buffalo.edu (S.D.M.); ram@compbio.org (R.S.)

check for updates

Citation: Bruggemann, L.; Falls, Z.; Mangione, W.; Schwartz, S.A.; Battaglia, S.; Aalinkeel, R.; Mahaja, S.D.; Samudrala, R. Supplementary Material: Multiscale Analysis and Validation of Effective Drug Combinations Targeting Driver Kras Mutations in Non-Small Cell Lung Cancer. *Int. J. Mol. Sci.* **2022**, *1*, 0. https://doi.org/

Academic Editors: Adam Jarmuła and Piotr Maj

Received: 15 September 2022
Accepted: 6 November 2022
Published: 30 November 2022

Publisher's Note: MDPI stays neutral with regard to jurisdictional claims in published maps and institutional affiliations.

Copyright: © 2022 by the authors. Licensee MDPI, Basel, Switzerland. This article is an open access article distributed under the terms and conditions of the Creative Commons Attribution (CC BY) license (https://creativecommons.org/licenses/by/4.0/).

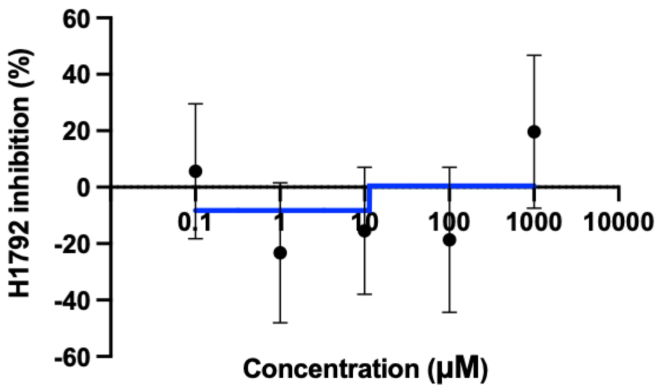

Figure S1. GI50 for BAY-293. The concentration (horizontal axis) of osimertinb is plotted against the H1792 cellular inhibition percentage (vertical axis). The GI50, or concentration required for 50% cellular inhibition, for BAY-293 was unable to be calculated with Graphpad prism 9.0, as there was not a strong enough effect. This indicates that BAY-293 is not effective at decreasing cellular proliferation in H1792 as a single agent.

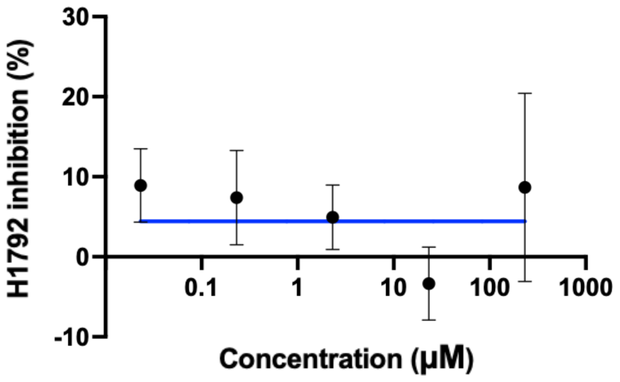

Figure S2. GI50 for ARS-1620. The concentration (horizontal axis) of osimertinb is plotted against the H1792 cellular inhibition percentage (vertical axis). The GI50, or concentration required for 50% cellular inhibition, for ARS-1620 was unable to be calculated with Graphpad prism 9.0, as there was not a strong enough effect. This indicates that ARS-1620 is not effective at decreasing cellular proliferation in H1792 as a single agent.

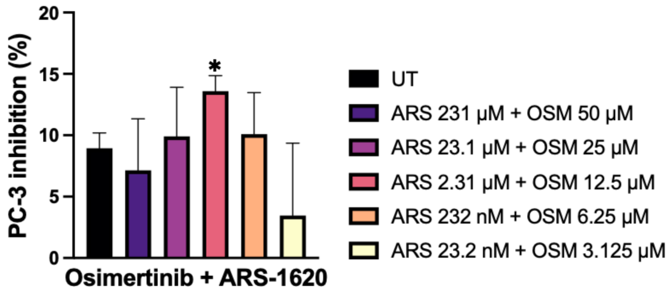

Figure S3. Cellular proliferation for PC-3 with ARS-1620 + osimertinib. The concentration (horizontal axis) of osimertinb and ARS-1620 is plotted against the PC-3 cellular inhibition percentage (vertical axis). The third strongest treatment condition was slightly significant ($p < 0.01$). This indicates that the osimertinib and ARS-1620 combination does not inhibit cellular proliferation in PC-3 relative to the untreated control.

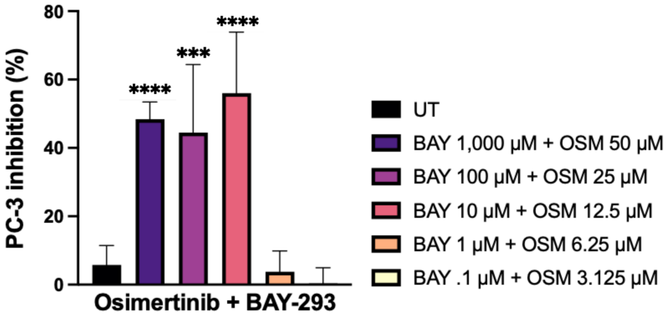

Figure S4. Cellular proliferation for PC-3 with BAY-293 + osimertinib. The concentration (horizontal axis) of osimertinb and BAY-293 is plotted against the PC-3 cellular inhibition percentage (vertical axis). The three strongest treatment conditions were all significantly higher ($p < 0.001$) compared to the untreated control. This indicates that while the osimertinib and BAY-293 combination shows some effect at inhibiting PC-3 cellular proliferation, it is far less than the effect observed with H1792.
