# Supplementary figures and images for: Multiscale Analysis and Validation of Effective Drug Combinations Targeting Driver KRAS Mutations in Non-Small Cell Lung Cancer
Source: Int J Mol Sci. 2023 Jan 5;24(2):997. doi: 10.3390/ijms24020997 (PMC9867122; doi:10.3390/ijms24020997)

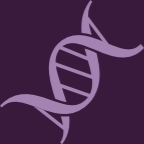

# International Journal of *Molecular Sciences*

Supplement: Supplementary file 1 [file ijms-24-00997-s001.zip › Definitions/ijms-logo-eps-converted-to.pdf]

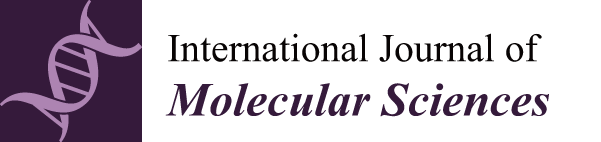

Supplement: Supplementary file 1 [file ijms-24-00997-s001.zip › Definitions/ijms-logo.png]

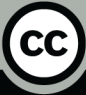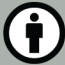

BY

Supplement: Supplementary file 1 [file ijms-24-00997-s001.zip › Definitions/logo-ccby-eps-converted-to.pdf]

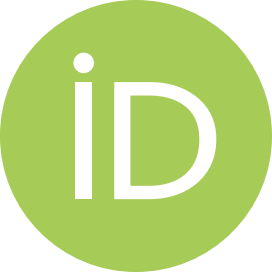

Supplement: Supplementary file 1 [file ijms-24-00997-s001.zip › Definitions/logo-orcid.pdf]

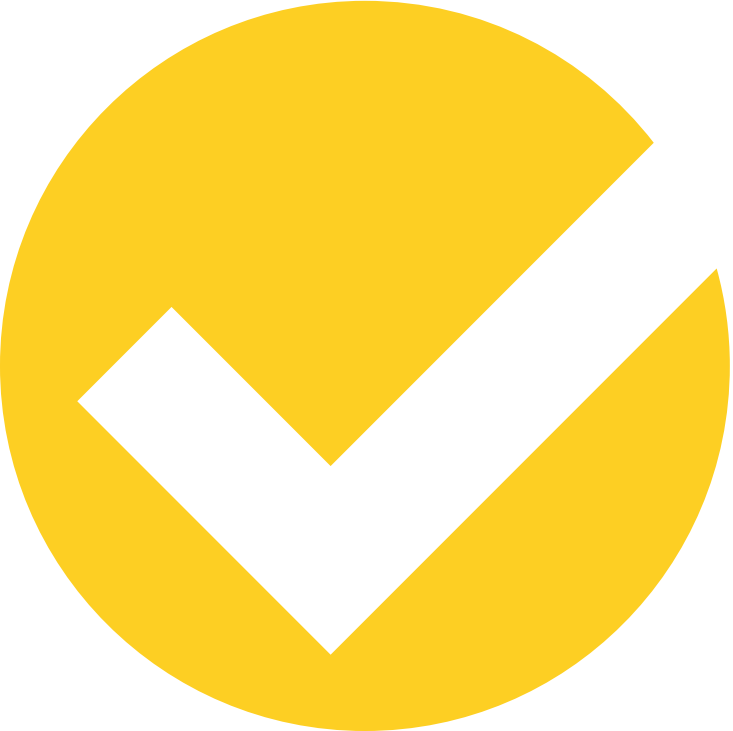

check for  
updates

Supplement: Supplementary file 1 [file ijms-24-00997-s001.zip › Definitions/logo-updates-eps-converted-to.pdf]

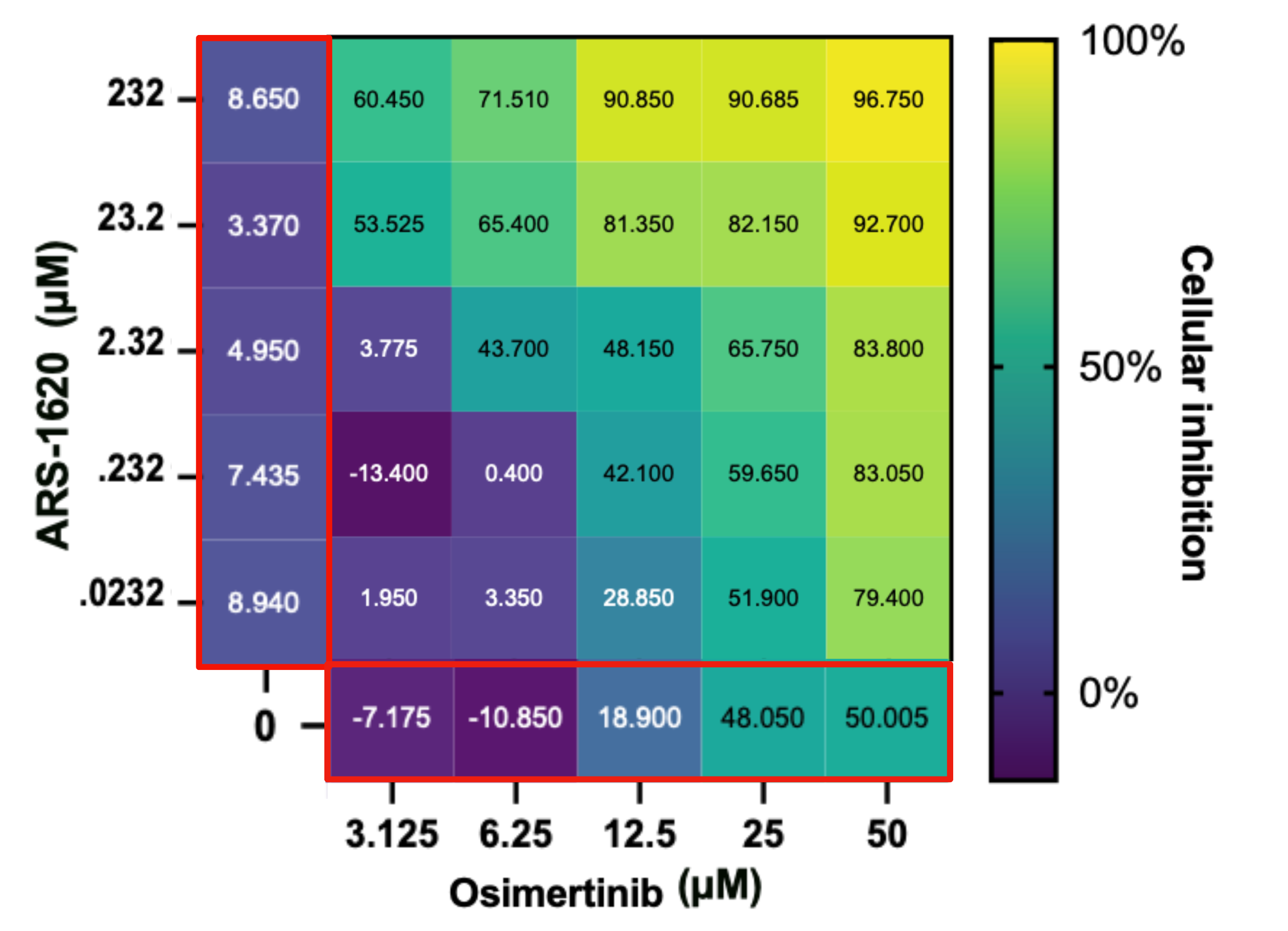

Supplement: Supplementary file 1 [file ijms-24-00997-s001.zip › figs/ARS Heatmap 2 (1).png]

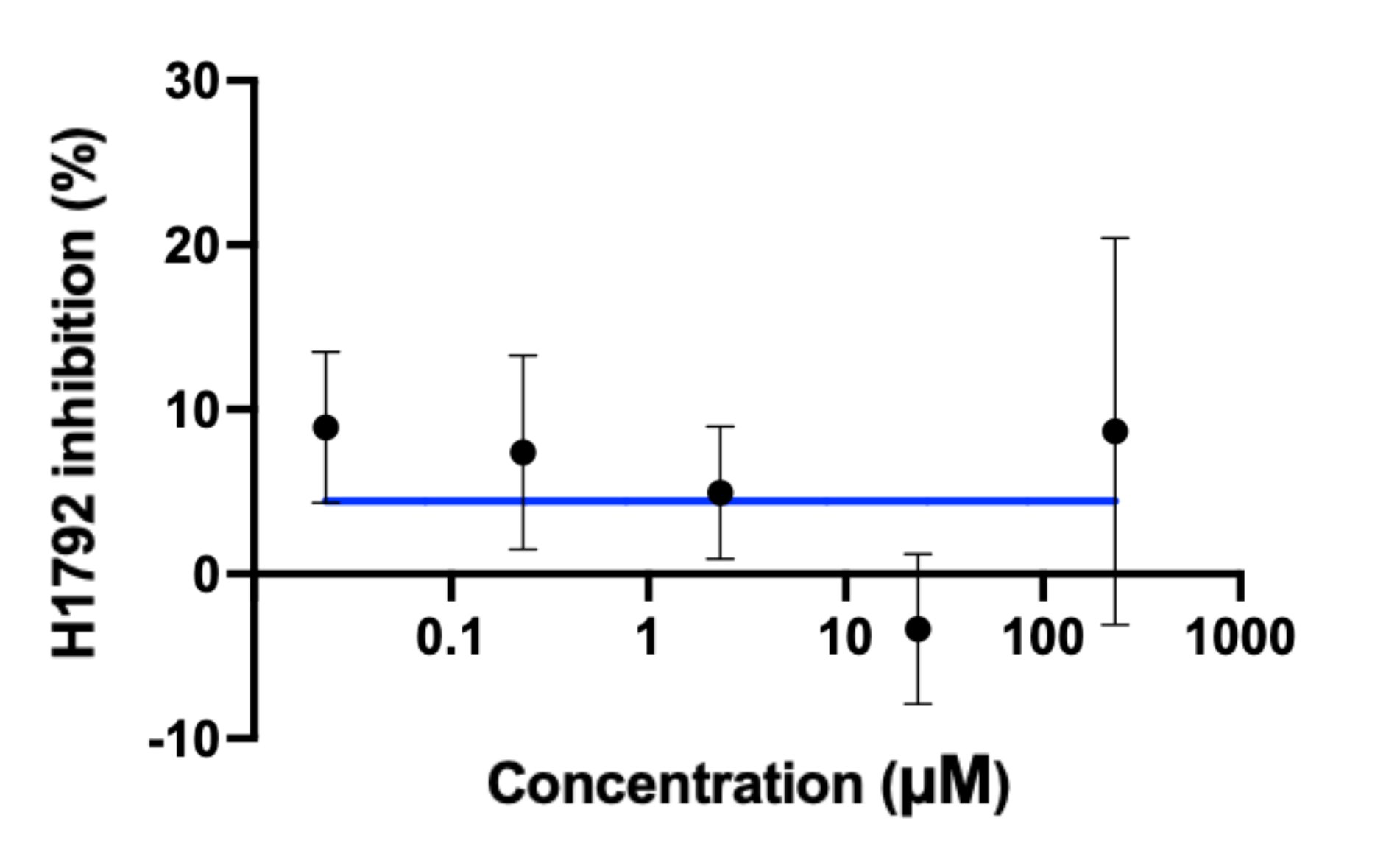

Supplement: Supplementary file 1 [file ijms-24-00997-s001.zip › figs/ARS inhibition.png]

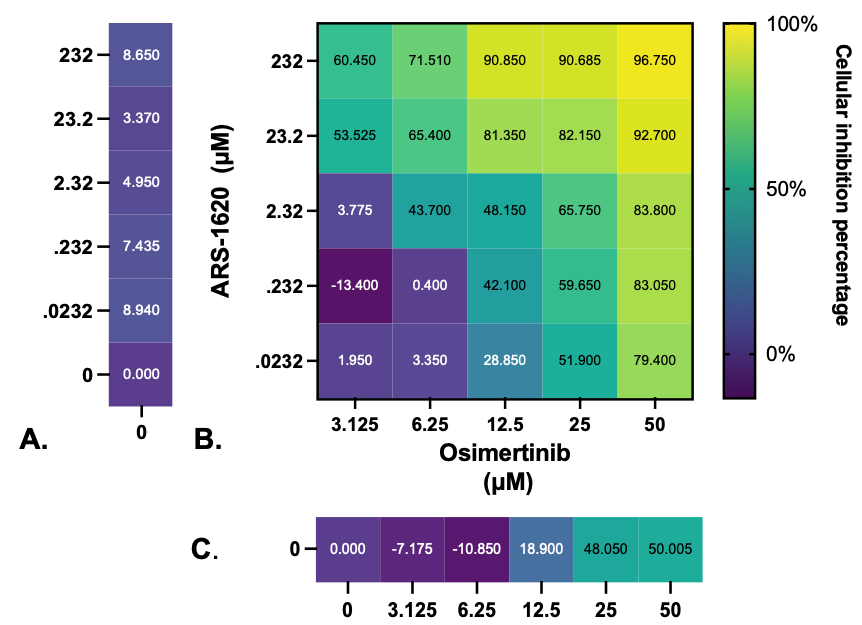

Supplement: Supplementary file 1 [file ijms-24-00997-s001.zip › figs/ARS+OSM.png]

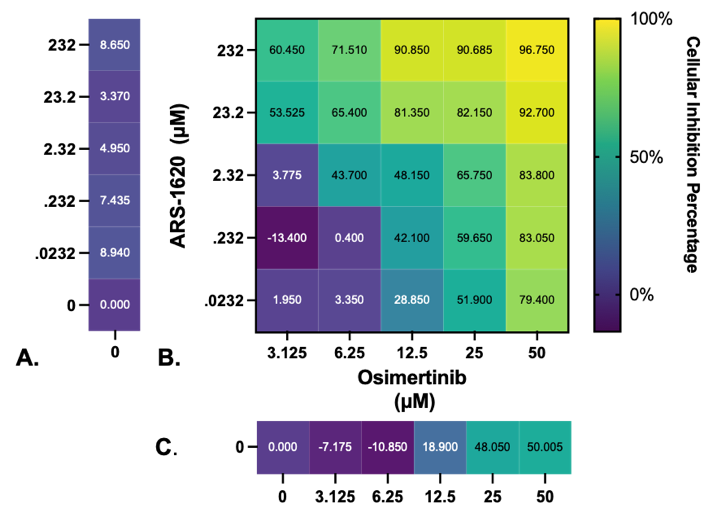

Supplement: Supplementary file 1 [file ijms-24-00997-s001.zip › figs/ARS_OSM heatmap.png]

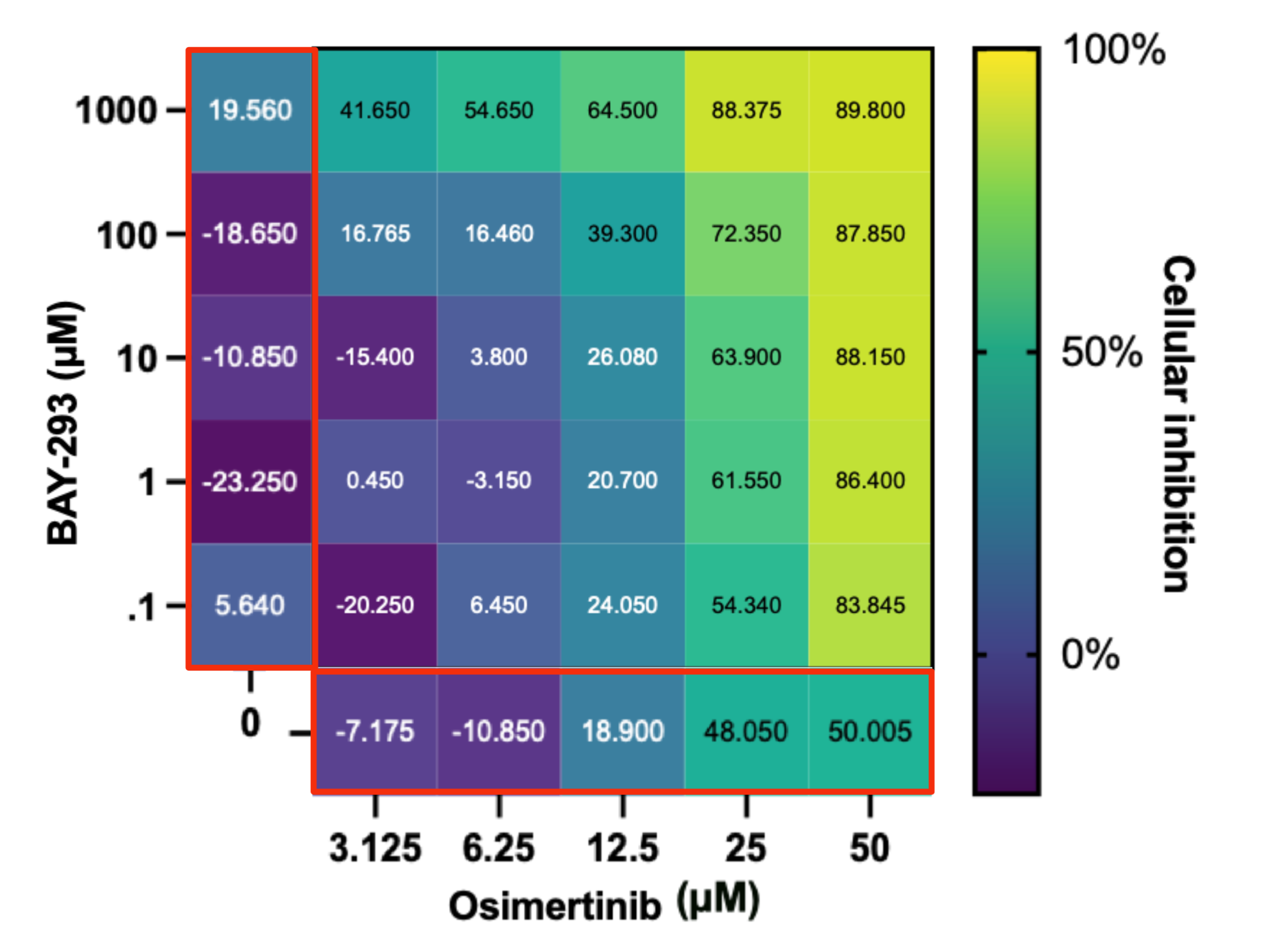

Supplement: Supplementary file 1 [file ijms-24-00997-s001.zip › figs/BAY heatmap 2.png]

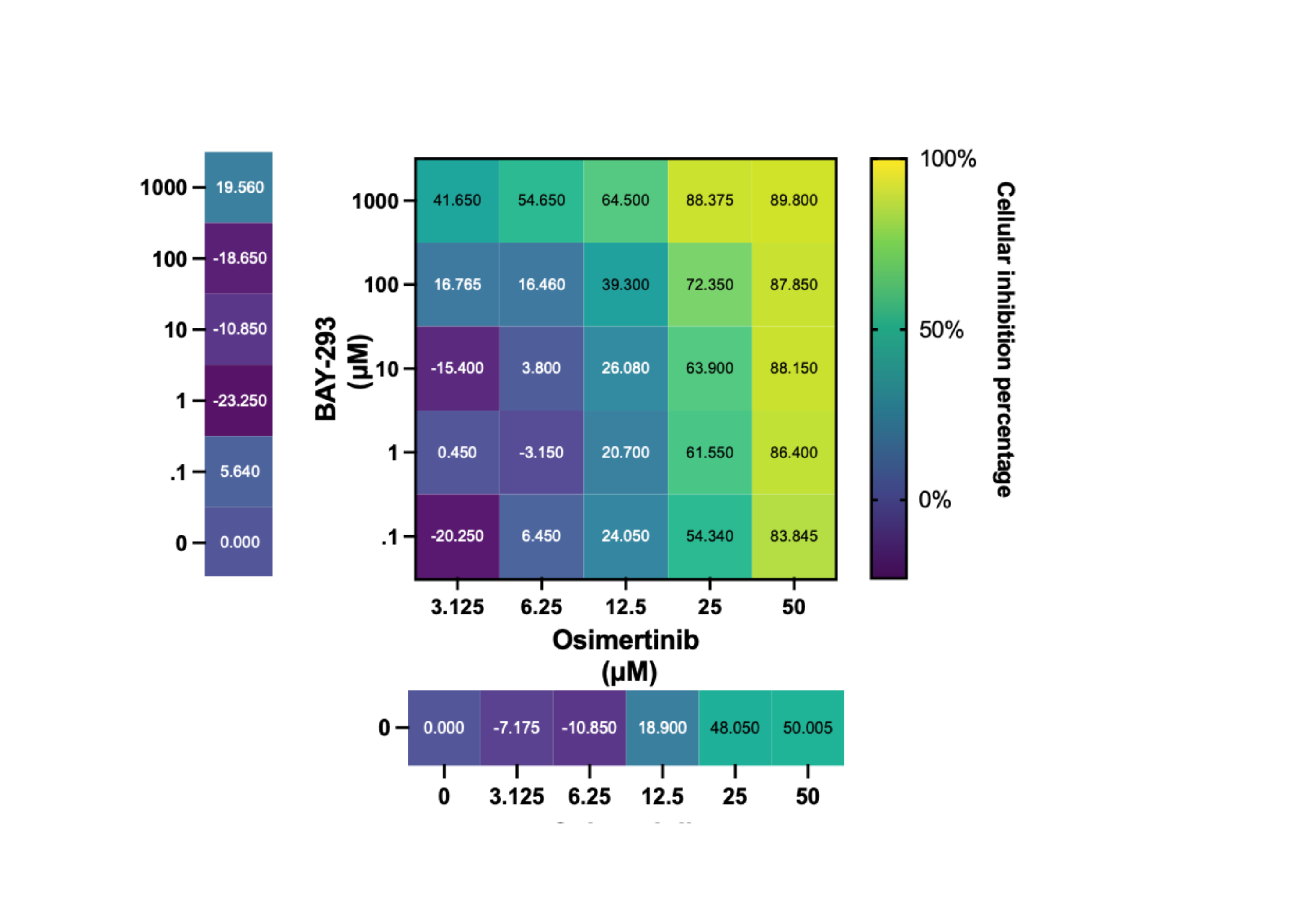

Supplement: Supplementary file 1 [file ijms-24-00997-s001.zip › figs/BAY heatmap.png]

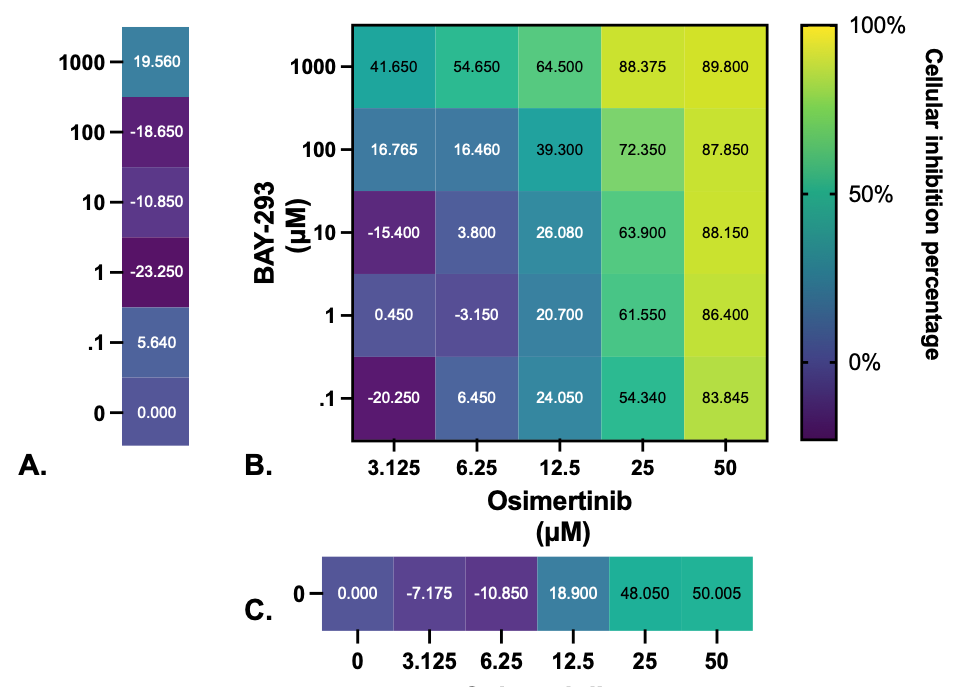

Supplement: Supplementary file 1 [file ijms-24-00997-s001.zip › figs/BAY+OSM.png]

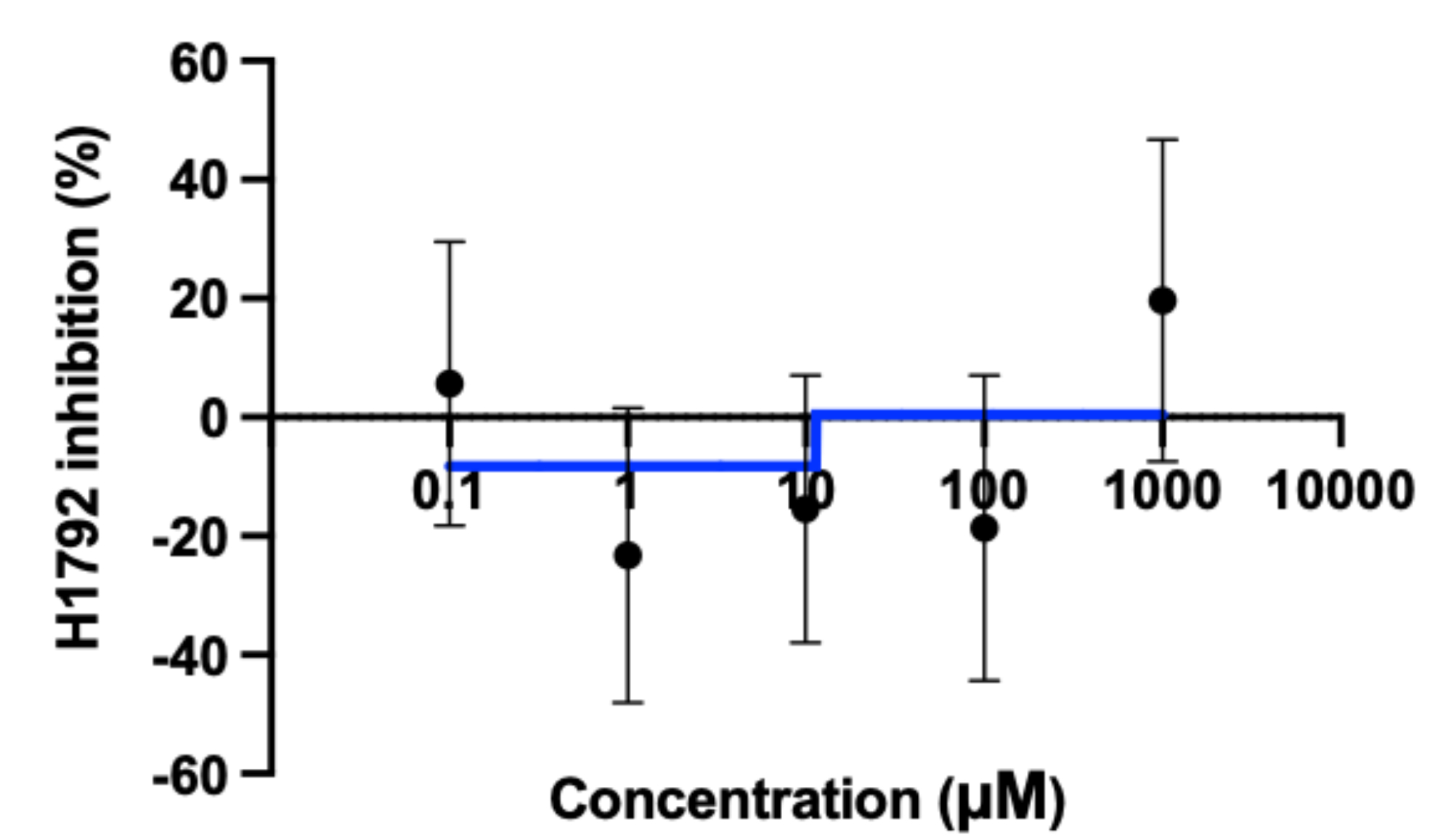

Supplement: Supplementary file 1 [file ijms-24-00997-s001.zip › figs/BAY-293.png]

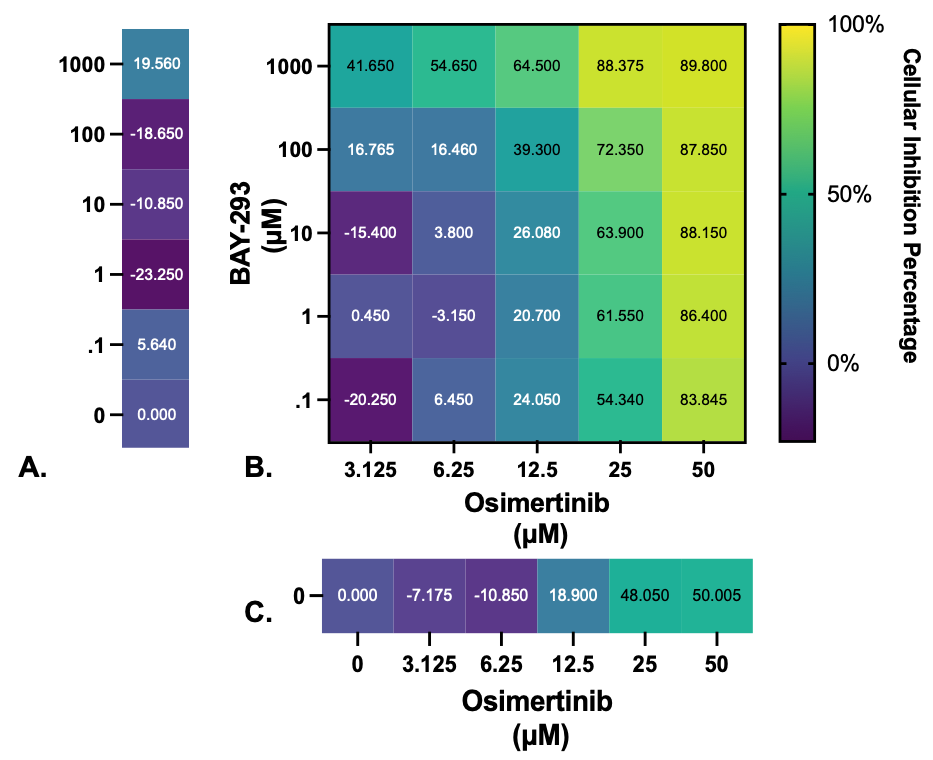

Supplement: Supplementary file 1 [file ijms-24-00997-s001.zip › figs/BAY-OSM.png]

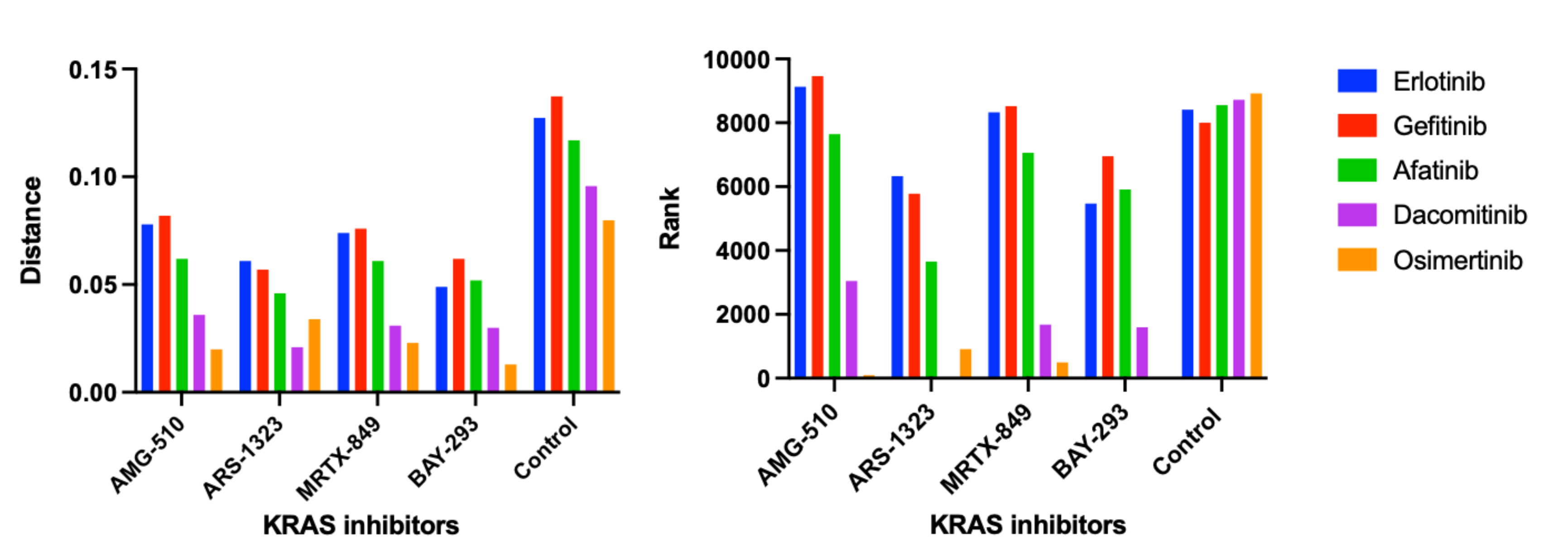

Supplement: Supplementary file 1 [file ijms-24-00997-s001.zip › figs/cosine distances.png]

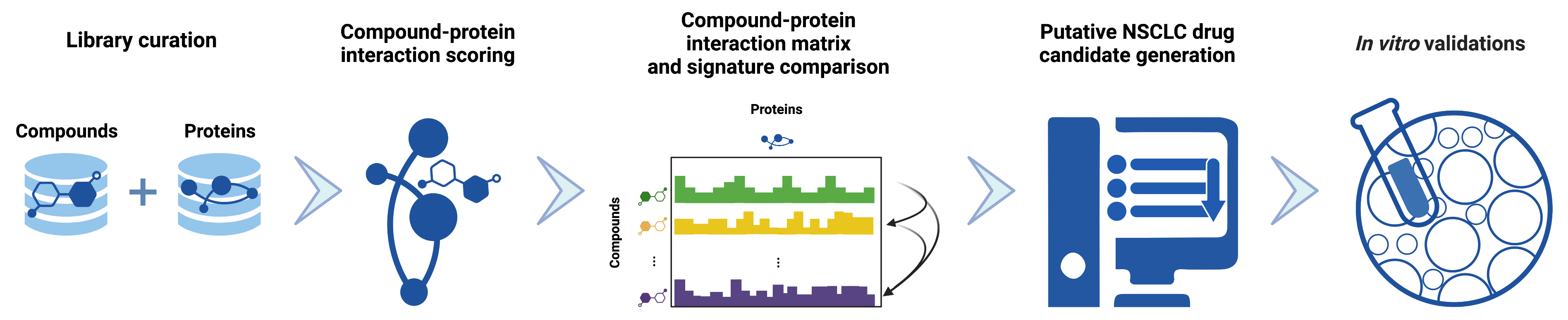

Supplement: Supplementary file 1 [file ijms-24-00997-s001.zip › figs/Drug Development Process (Layout).png]

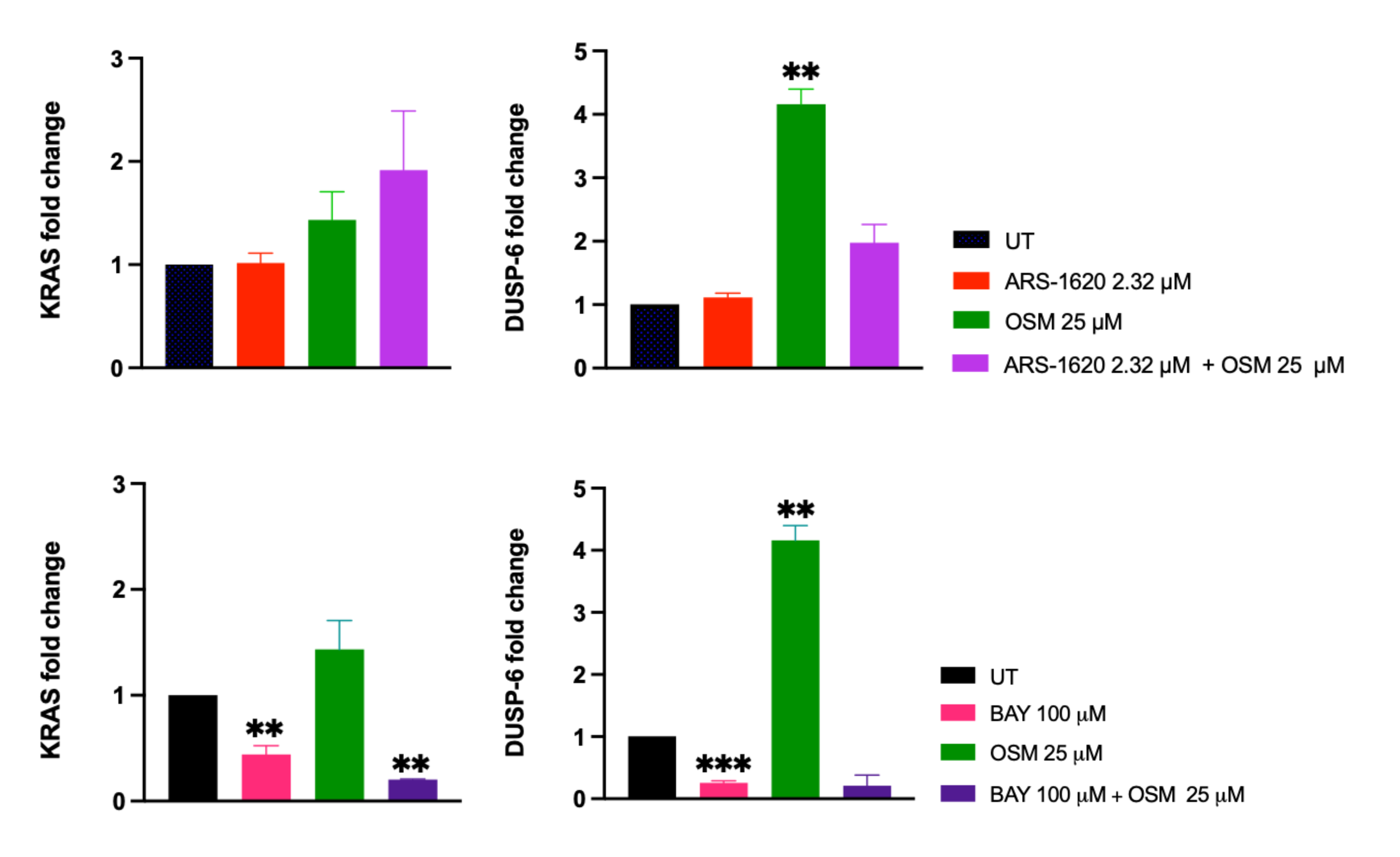

Supplement: Supplementary file 1 [file ijms-24-00997-s001.zip › figs/GE data updated.png]

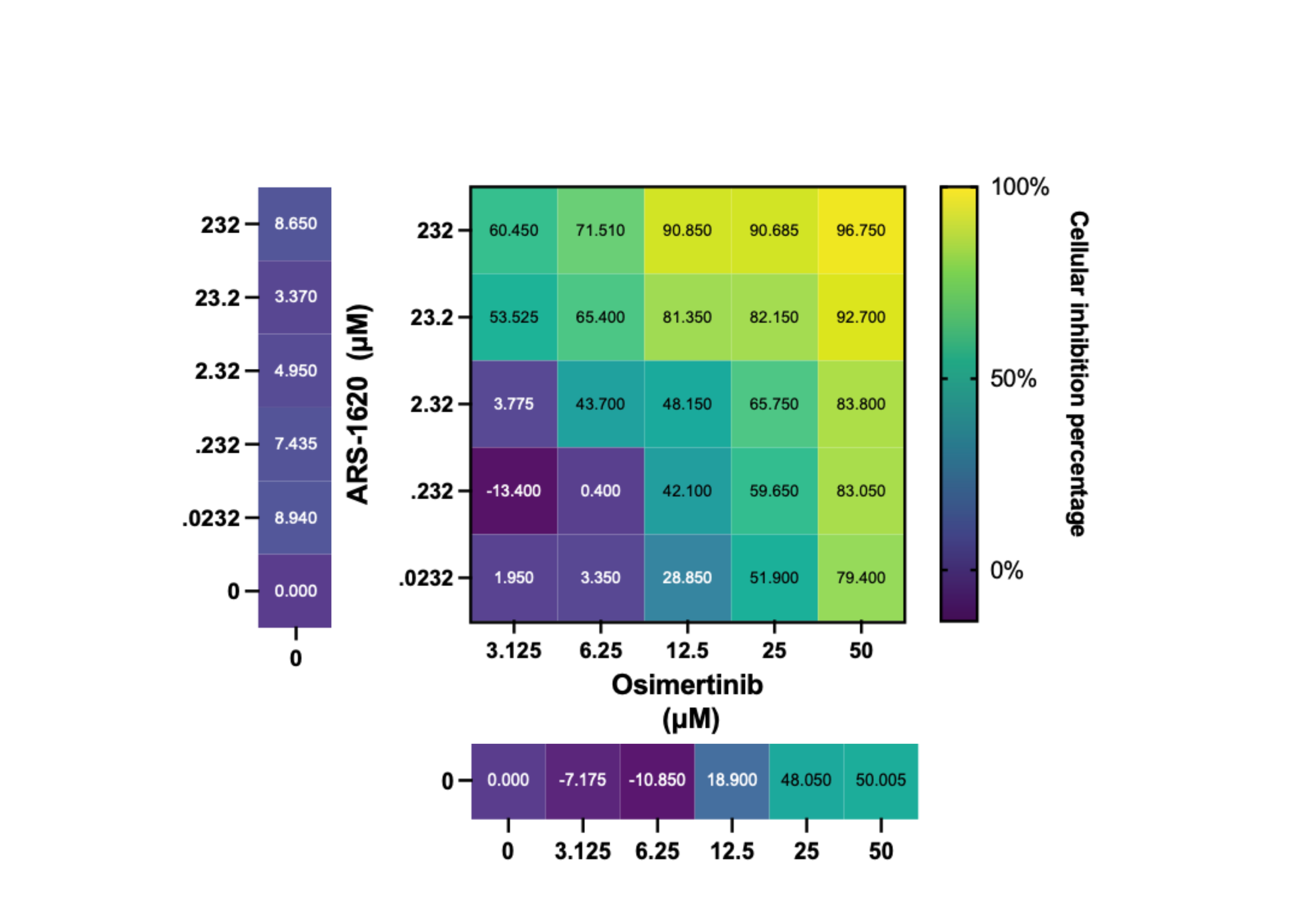

Supplement: Supplementary file 1 [file ijms-24-00997-s001.zip › figs/heat map ARS.png]

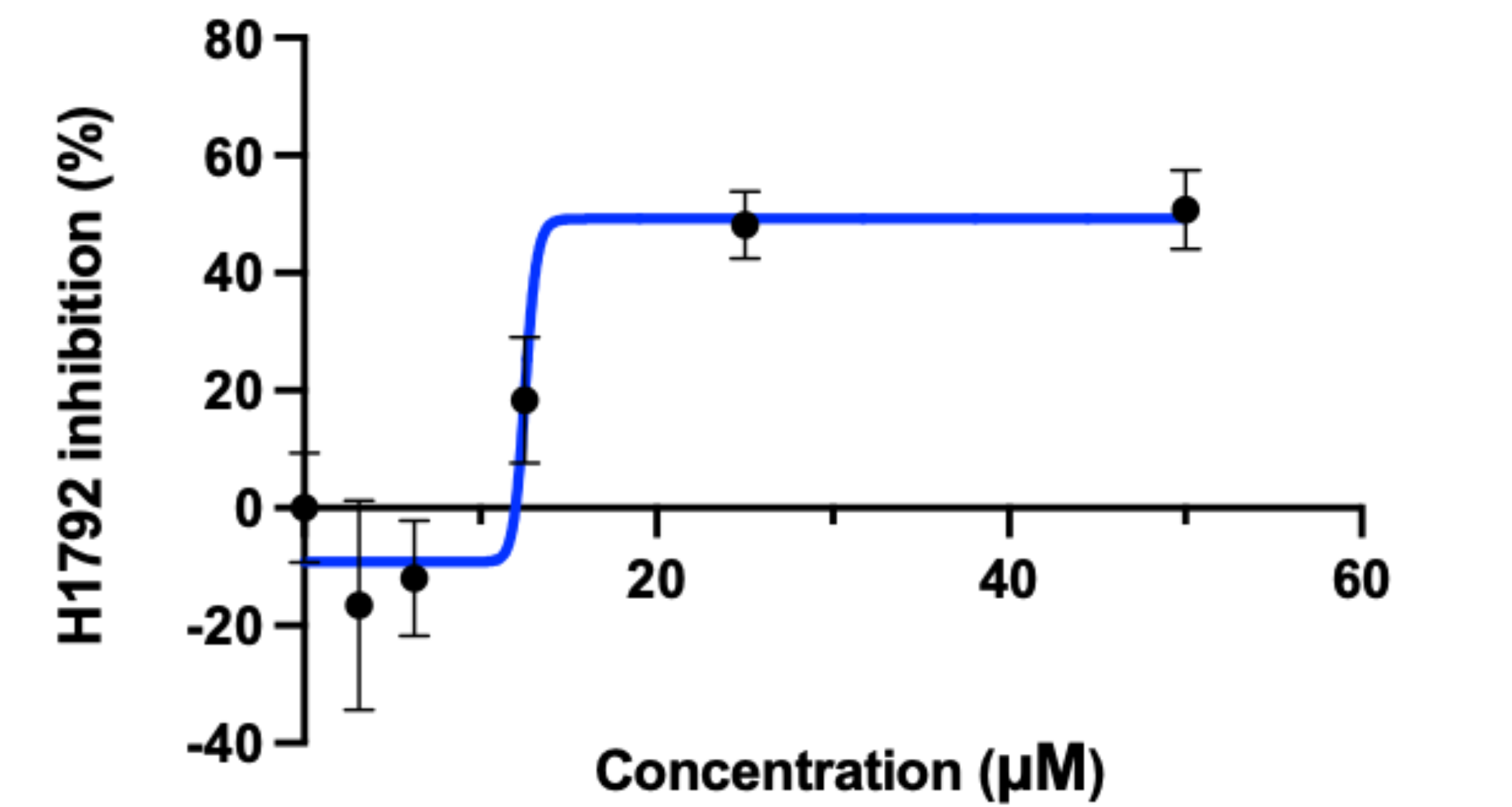

Supplement: Supplementary file 1 [file ijms-24-00997-s001.zip › figs/OSM.png]

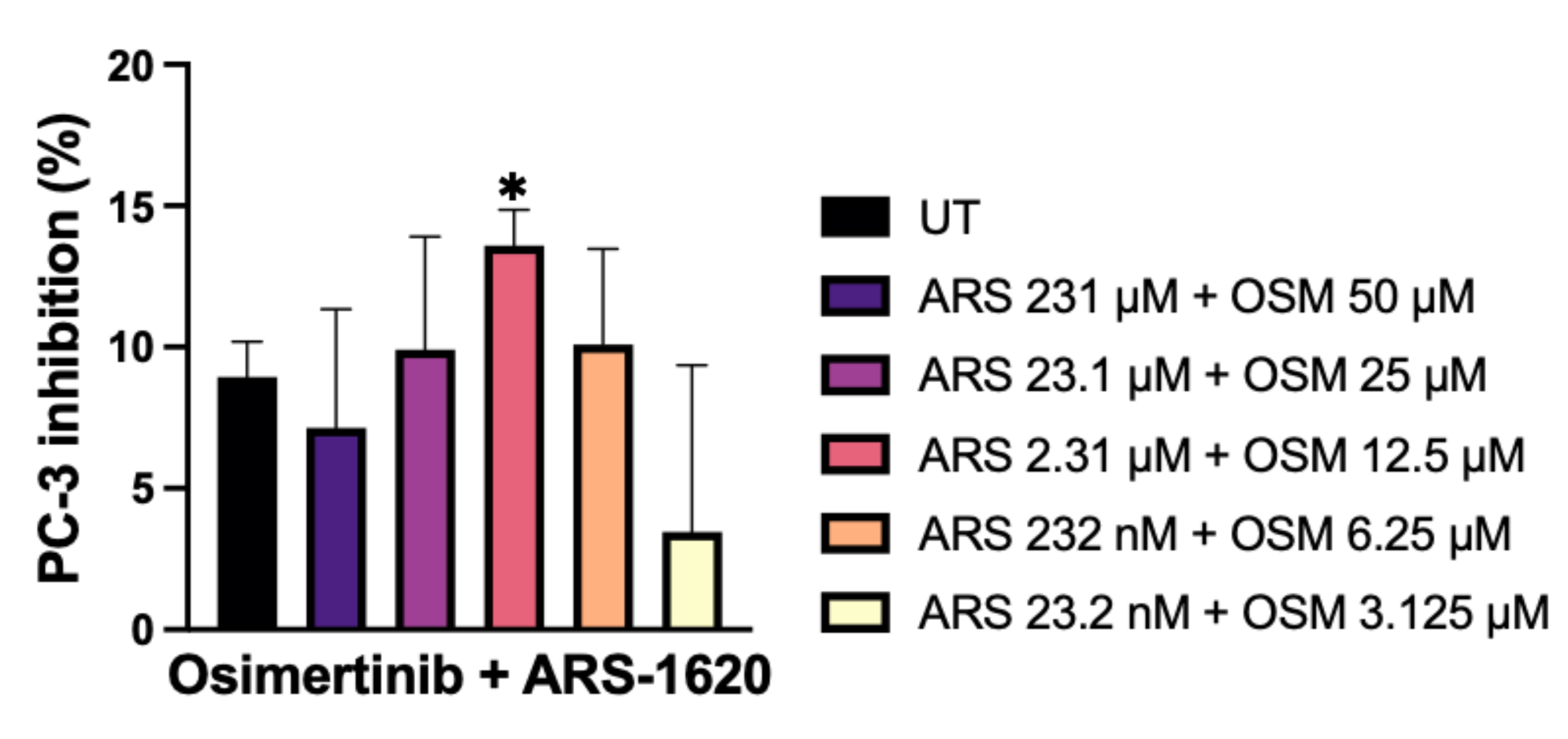

Supplement: Supplementary file 1 [file ijms-24-00997-s001.zip › figs/PC-3 ARS+OSM.png]

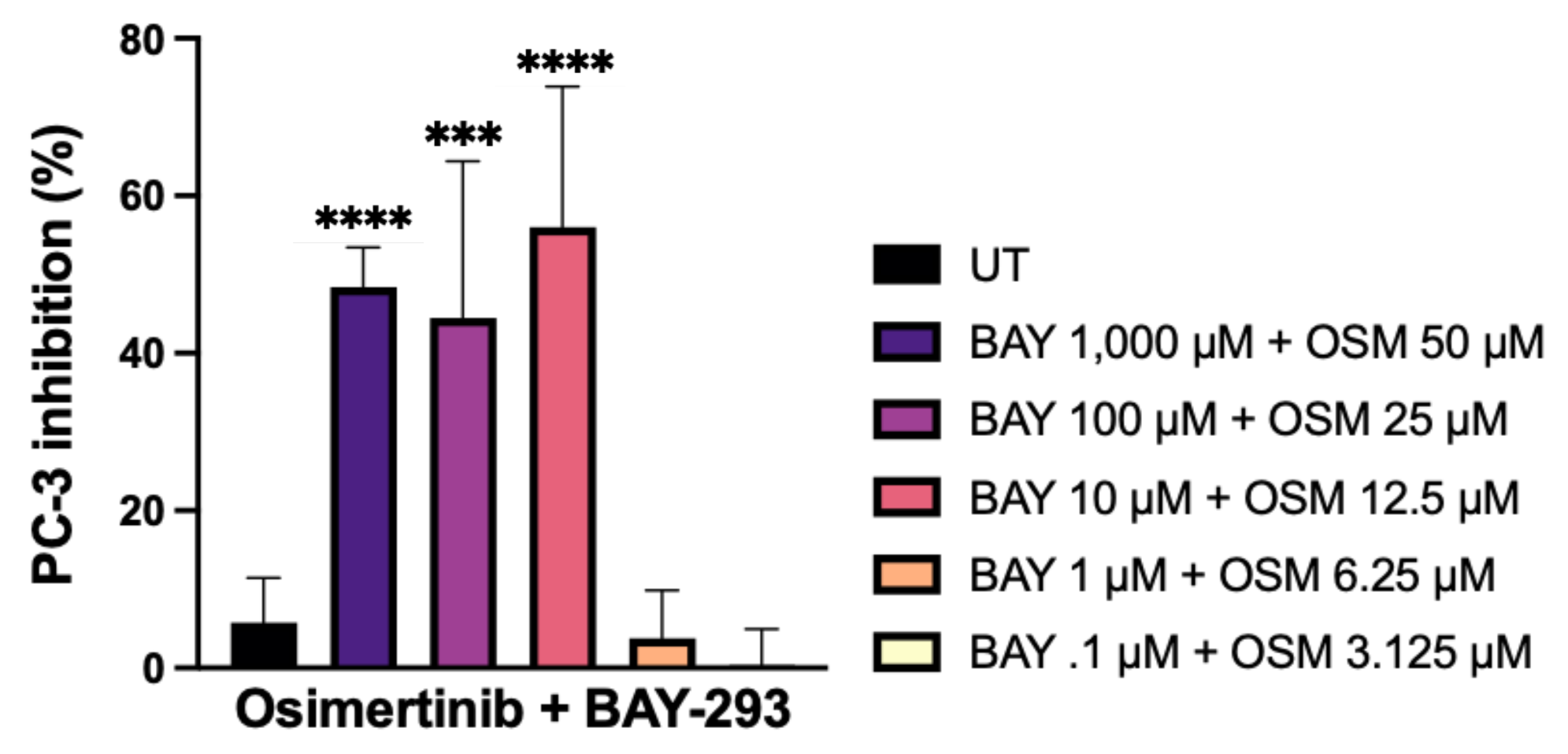

Supplement: Supplementary file 1 [file ijms-24-00997-s001.zip › figs/PC-3 BAY+OSM.png]

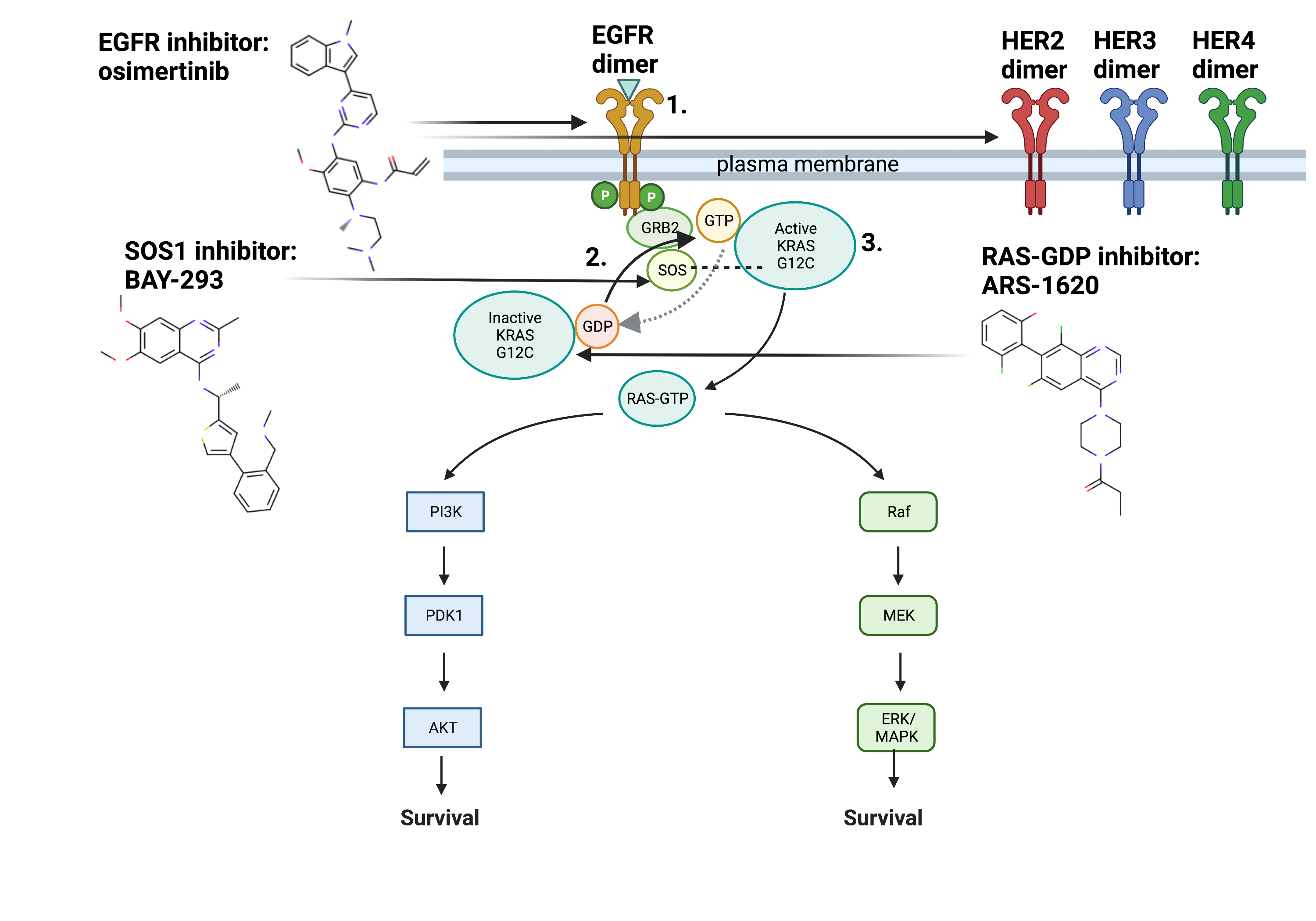

Supplement: Supplementary file 1 [file ijms-24-00997-s001.zip › figs/Ras Pathway.png]
